# Gyriform restricted diffusion in adults: looking beyond thrombo-occlusions

**DOI:** 10.1186/s13244-019-0829-0

**Published:** 2020-02-10

**Authors:** Vivek Pai, Yih Yian Sitoh, Bela Purohit

**Affiliations:** Department of Neuroradiology, National Neuroscience Institute, 11 Jalan Tan Tock Seng, Singapore, 308433 Singapore

**Keywords:** Gyrifom, Restricted diffusion, Non-vascular aetiologies

## Abstract

Gyriform restricted diffusion (GRD) refers to hyperintense signal involving the cerebral cortex on diffusion-weighted images (DWI) with corresponding hypointensity on apparent diffusion coefficient (ADC) images. These changes are commonly seen following a vascular occlusion, reflecting the limitation of water molecule movement across cell membranes (restricted diffusion) due to the failure of Na^+^/K^+^-ATPase pumps (cytotoxic oedema). However, GRD can occur in several other neurological conditions as well. A thorough understanding of these conditions and their anatomic predilection plays a critical role in identifying and differentiating them from vascular thrombo-occlusion, with impact towards appropriate clinical management. This review highlights the less commonly encountered, non-stroke causes of GRD in adults with case-based examples. A tabulated chart of the patterns of cortical and subcortical involvement associated with these aetiologies is provided for a quick, pattern-based reference for daily radiological reporting.

## Teaching points

- Causes of GRD include, but are not limited to, vascular occlusion.
- Non-stroke GRD in adults may be due to haemodynamic, metabolic, infectious and rarely genetic causes.
- These conditions pose a diagnostic challenge and vary in treatment and outcomes.
- Identifying patterns of GRD and additional involvement of other anatomic structures often helps to diagnose the aetiology.
- GRD related to some aetiologies is reversible.

## Introduction

Diffusion-weighted imaging (DWI) is one of the most robust imaging sequences used in MRI especially in clinical neuroradiology. Since its advent in the mid-1980s, DWI has been the mainstay in acute stroke imaging. In acute brain parenchymal ischaemia following vascular thrombo-occlusion, failure of sodium-potassium adenosine triphosphate (ATP)-dependent (Na^+^/K^+^-ATPase) pumps forms the premise of cytotoxic oedema or accumulation of water within neurons. The lack of transmembrane motion of water, in other words ‘restriction’ of their random Brownian motion, causes hyperintense signal on DWI and hypointense signal on apparent diffusion coefficient (ADC) images [1–3]. These changes initially affect the grey matter due to its high metabolic activity and high astrocyte density [2].

Apart from a vascular thrombo-occlusive process causing restricted diffusion in the cortical grey matter, several other aetiologies may result in a similar pattern of GRD [4]. Prompt identification of these other pathologies is vital for appropriate patient management and ensuring a favourable outcome. To the best of our knowledge, there is limited literature comprehensively describing the clinico-radiological features of these conditions. This review focuses on identifying and describing non-stroke causes of GRD in adults with the aim to educate the trainee radiologist, especially as these cases are often read as urgent/on-call scans. We have provided multiple imaging examples and a table describing a pattern-based approach to these conditions, to facilitate learning.

Broad overview of non-stroke causes of GRD in adults:

1. Haemodynamic alterations: Hypoxic-ischaemic encephalopathy, post-ictal changes
2. Metabolic causes: Hypoglycaemia, hyperammonaemia
3. Infections: Herpes encephalitis, Creutzfeldt-Jakob Disease (CJD), cerebritis
4. Genetic: MELAS (Mitochondrial myopathy, encephalopathy, lactic acidosis, stroke-like episodes)

## Haemodynamic alterations: hypoxic-ischaemic encephalopathy

Hypoxic-ischaemic encephalopathy (HIE) is a devastating neurological condition, often leading to death or severe neurological disability. It is caused by a myriad of aetiologies, including cardiac arrest, hypotension, trauma, drowning and asphyxiation. As the name suggests, the common pathophysiological events leading to HIE include global reduction of cerebral blood supply and low blood oxygen tension [5, 6].

In adults, HIE primarily affects grey matter structures (selective neuronal necrosis). This selective vulnerability of grey matter is due to its high metabolic activity and presence of abundant dendrites and capillaries. At the molecular level, prolonged anoxia converts oxidative phosphorylation to anaerobic metabolism. This leads to depletion of ATP, increased lactate, cytotoxic oedema and cell death. These changes limit the transmembrane motion of water, thus producing hyperintense signal in the grey matter on DWI [5–7].

**Fig. 1:**
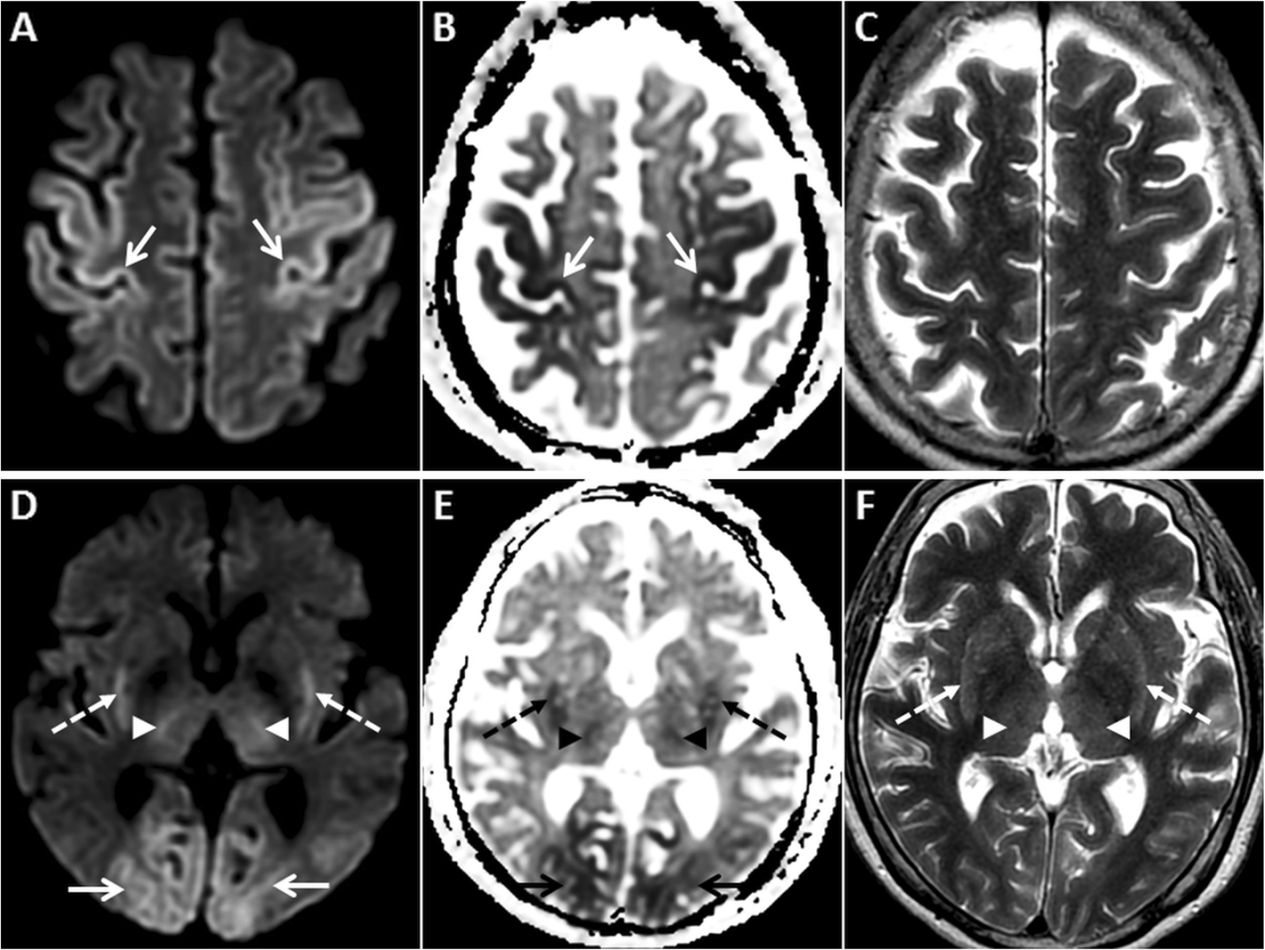
MRI of the brain performed in the acute phase of HIE. On axial DWI (**a**, **d**) and axial ADC images (**b**, **e**), symmetric GRD is seen in the perirolandic cortices (arrows in **a** and **b**) and in the visual cortices bilaterally (arrows in **d** and **e**). Restricted diffusion is also observed in the putamina (dashed arrows in **d** and **e**) and in the thalami (arrowheads in **d** and **e**). Corresponding axial T2W images (**c**, **f**) demonstrate no obvious signal abnormality in the cortices. However, there is mild T2 hyperintensity in the putamina (dashed arrows) and in the thalami (arrowheads) (**f**)

**Fig. 2:**
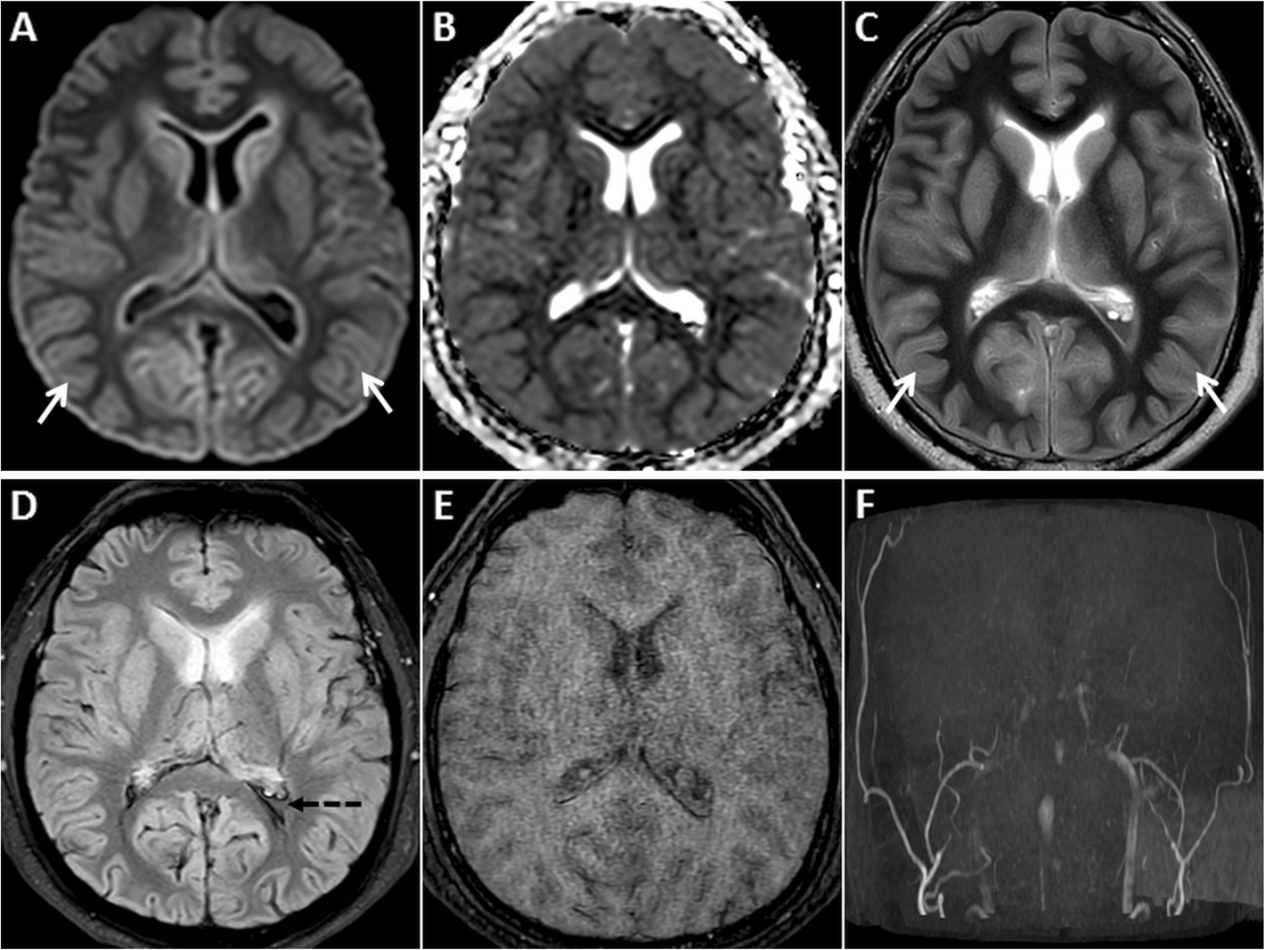
MRI of the brain performed in the subacute phase of HIE. DWI hyperintensity is noted in the deep grey nuclei and extensively in the cerebral cortices (arrows) on axial DWI image (**a**). There is no signal drop on axial ADC image (**b**). Diffuse cortical swelling and signal changes (arrows) are appreciated better on the axial T2W image (**c**). Minimal intraventricular blood (dashed arrow) is seen on axial GRE image (**d**). Axial source 3D-TOF MRA image (**e**) and reconstructed maximum intensity projection MRA image of Circle of Willis (**f**) show no flow signal in the intracranial arteries

MRI is vital for diagnosing HIE and identifying the age of insult. DWI is the first imaging sequence to show the changes of HIE (within 1 h). Restricted diffusion is typically seen in the cerebral cortex (especially perirolandic and visual cortices), deep grey nuclei (basal ganglia and thalami), cerebellum and hippocampi (Fig. 1). DWI hyperintense signals generally fade by the end of the first week, a phenomenon known as pseudonormalisation (Fig. 2). During this early subacute phase (24 h–2 weeks), injured grey matter structures show T2 hyperintensity and swelling [5–7]. Gyrifom enhancement of the injured cortex may be seen in this phase [8, 9]. In the chronic stage, T1 hyperintense changes of cortical necrosis are seen along with residual T2 hyperintensity and gliosis in the basal ganglia [5].

## Haemodynamic alterations: post-ictal changes

MRI following a single seizure or status epilepticus may often show GRD. Seizures induce changes in neuronal activity, vascular tone and blood oxygenation resulting in a mismatch between brain energy delivery and consumption. Hyperperfusion occurs due to increased glucose and oxygen metabolism. Eventually anaerobic metabolism ensues, resulting in lactic acidosis, depletion of energy substrates and failure of Na^+^/K^+^-ATPase pump leading to cytotoxic oedema. This resultant cytotoxic oedema and selective vulnerability of the grey matter explains the GRD. It is important to note that seizure-related restricted diffusion is often reversible, thereby indicating that cell death is not inevitable and that the causative pathophysiological pathways are possibly different from those of ischaemic infarction [3, 4, 10–13].

**Fig. 3:**
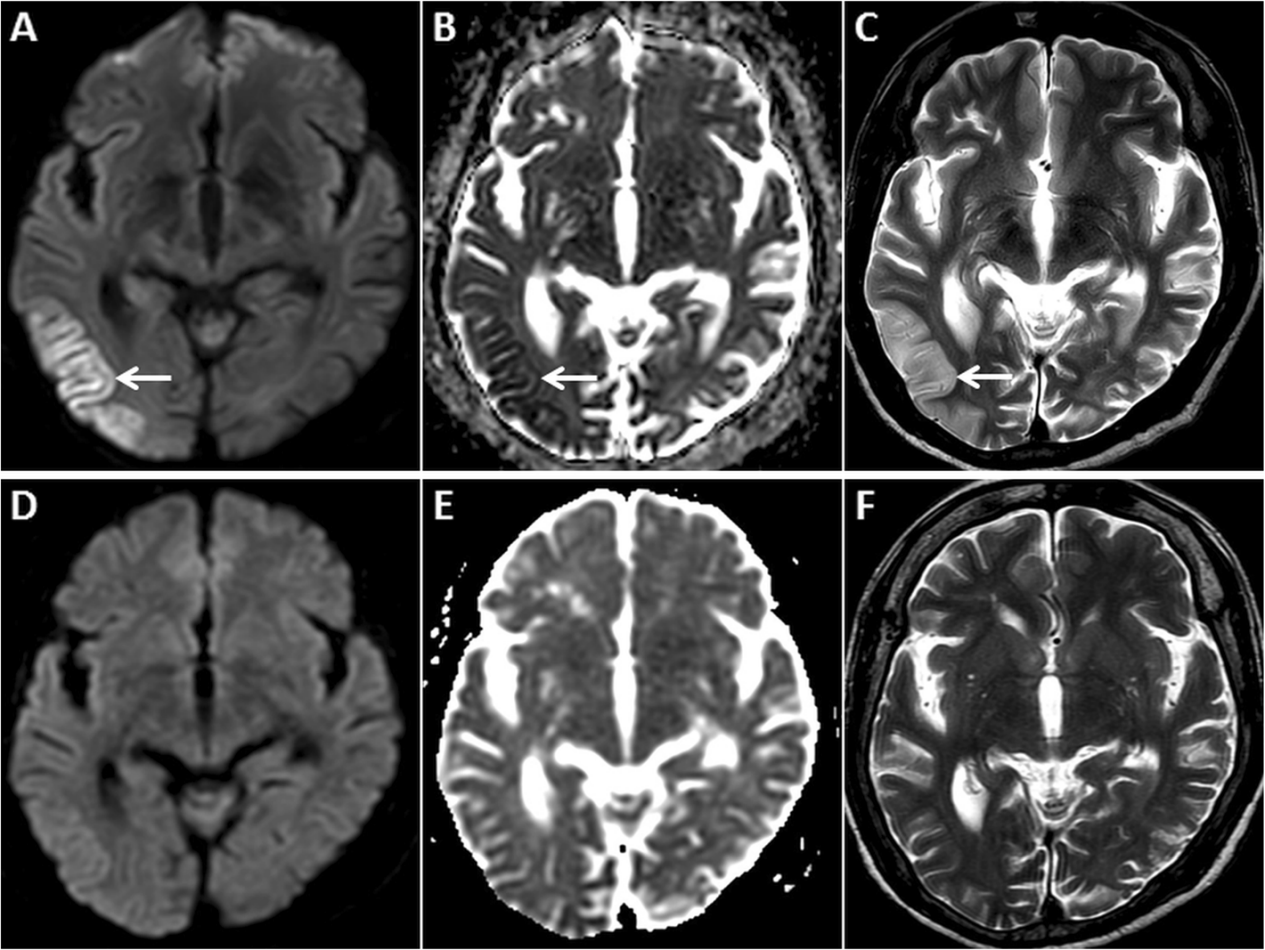
MRI of the brain in a 67-year-old female patient presenting with a seizure. GRD is observed in the right temporo-occipital region (arrow) on axial DWI (**a**) and ADC image (**b**). Also note associated cortical swelling and hyperintensity (arrow) on axial T2W image (**c**). Follow-up MRI after 4 days reveals complete resolution of the cortical signal abnormality and swelling on axial DWI, ADC and axial T2W images (**d**, **e**, **f**, respectively)

Post-seizure MRI often shows restricted diffusion in the hippocampi and/or in any of the cerebral cortices (Fig. 3). Unilateral or bilateral involvement may be seen, not corresponding to any vascular territory or boundary. GRD is often associated with gyral/cortical swelling and T2/ FLAIR hyperintensity [3, 4, 10–12]. Reversible/transient restricted diffusion has also been described in the basal ganglia and in the splenium of the corpus callosum [12, 13]. Contrast enhancement may occur due to a local increase in metabolism, vasodilation or a blood-brain barrier breach [12]. DWI signal abnormalities have been shown to correspond to areas of hypermetabolism on positron-emission tomography and maximum ictal electro-encephalographic (EEG) activity [4]. Serial post-ictal MRI studies may show either persistence or resolution of DWI signal abnormalities and sometimes cortical volume loss/gliosis [12].

Having discussed seizure-related GRD in detail, it is important to remember that the primary indication of brain MRI in post-ictal patients is to investigate the cause of seizures such as space-occupying lesions (e.g. neoplasms) or structural lesions (e.g. mesial temporal sclerosis). In this context, it is vital to not miss a focal parenchymal abnormality characterised by T2 hyperintensity (with/without focal restricted diffusion), mass effect, perilesional vasogenic oedema and/or enhancement which indicates the true causative pathology of the seizure [12].

## Metabolic causes: hypoglycaemia

The brain relies heavily on an uninterrupted supply of glucose for optimal functioning. Neurons have a rapid metabolic rate and can neither generate nor store significant amounts of glucose. Hence, sudden decline in blood glucose can jeopardise neuronal homeostasis within minutes. The neurological symptoms of hypoglycaemic encephalopathy typically occur at blood glucose levels less than 2.9 mmol/L. These include altered mentation, confusion, lethargy and seizures. If left untreated, these can progress to drowsiness, coma and even death. Common causative factors of severe hypoglycaemia are insulin/oral hypoglycaemic drug overdose, insulinomas, sepsis and alcohol abuse [14–17].

At the molecular level, hypoglycaemia arrests protein synthesis, leading to intracellular calcium influx and release of aspartate. Aspartate is a neurotoxin which is known to cause preferential neuronal necrosis in the cerebral cortex, neostriatum and hippocampus. Additionally, hypoglycaemia also causes an increase in the concentration of extracellular glutamate, which promotes the influx of calcium and sodium into cells inducing apoptosis [14–17].

**Fig 4:**
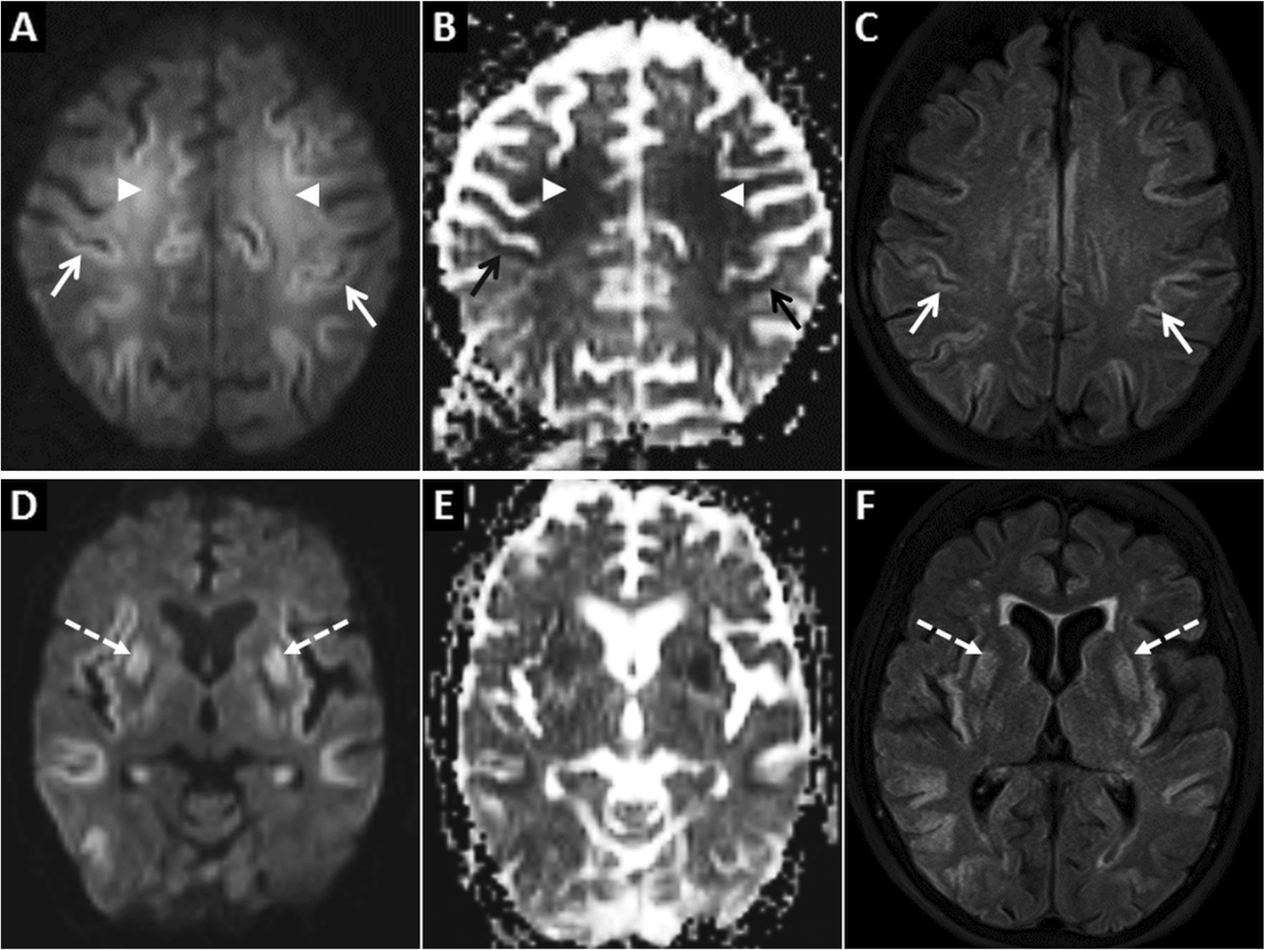
MRI of the brain in a 68-year-old male patient presenting with severe hypoglycaemia. Extensive symmetric GRD (arrows) and restricted diffusion in deep white matter (arrowheads) is seen on axial DWI (**a**) and ADC image (**b**). Restricted diffusion is also seen in the putamina bilaterally (dashed arrow) on axial DWI (**d**) and ADC image (**e**). Axial FLAIR images (**c**, **f**) show hyperintense signal in the involved cortex (arrows) and in the putamina (dashed arrows)

**Fig. 5:**
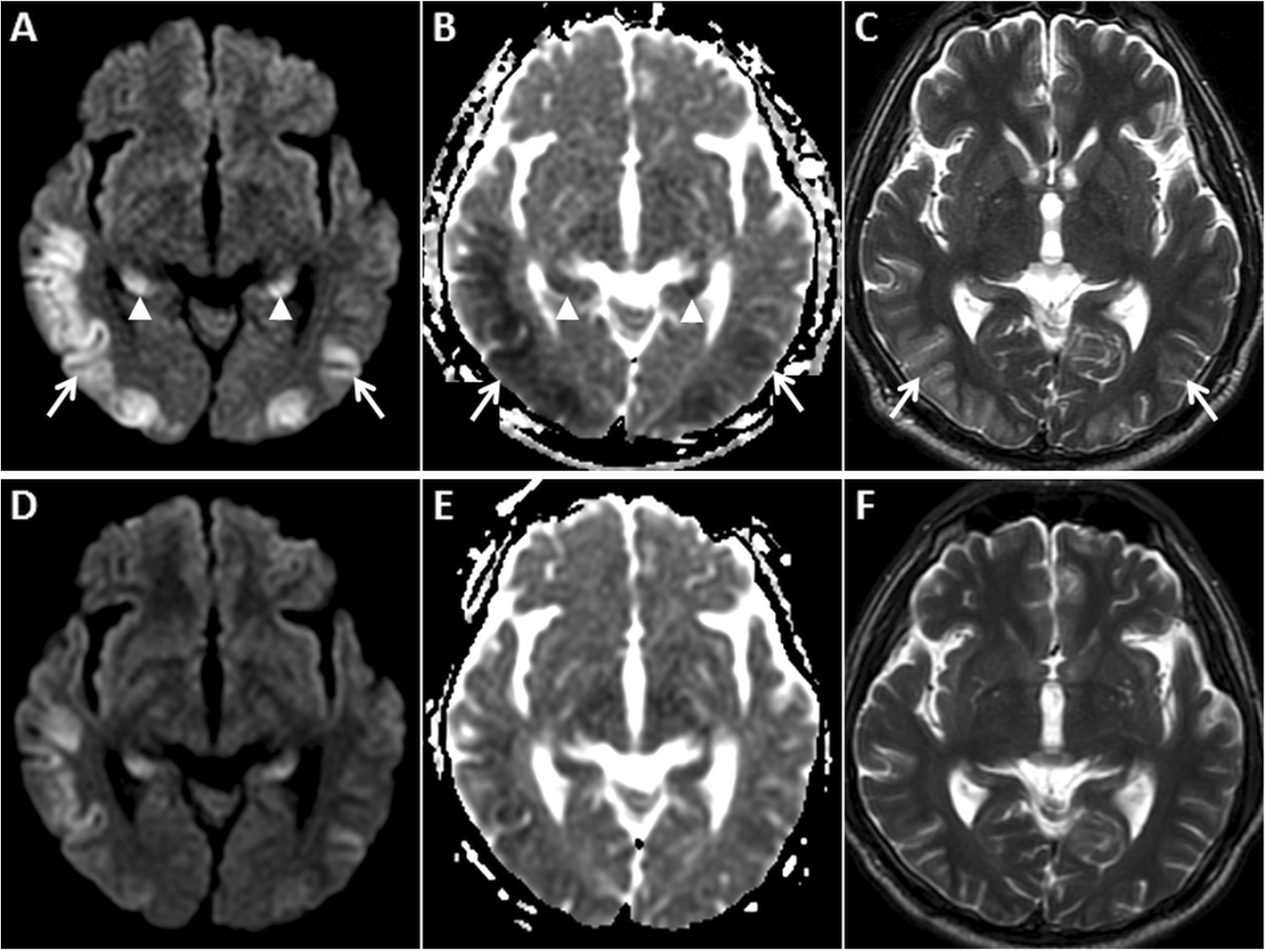
MRI of the brain in a 46-year-old male patient diagnosed with hypoglycaemia on the background of diabetic ketoacidosis. Bilateral asymmetric GRD, more on the right, is seen in the temporo-occipital lobes (arrows) on axial DWI (**a**) and ADC image (**b**). Restricted diffusion is also seen in bilateral hippocampi (arrowheads). Mild hyperintensity is noted in the involved cortex (arrows) on the axial T2W image (**c**). Post-treatment follow-up MRI after 2 weeks shows partial resolution of signal changes on axial DWI and ADC images (**d**, **e**, respectively). Note that the axial T2W image also appears almost unremarkable (**f**)

MRI is the imaging modality of choice for the diagnosis of hypoglycaemic encephalopathy and to determine prognosis. The earliest changes are seen on DWI with the involved areas showing restricted diffusion. On the basis of topographic distribution of signal abnormalities, common imaging patterns include (1) predominant grey matter involvement (cortex, neostriatum and hippocampus), (2) predominant white matter involvement (internal capsules, splenium of corpus callosum) and (3) mixed grey and white matter involvement [16] (Figs. 4 and 5).

DWI signal abnormalities may be unilateral or bilateral and may also show corresponding T2/FLAIR hyperintensity. Haemorrhages have not been reported in hypoglycaemia although contrast enhancement may occur [16, 17]. Patients with extensive cortical and white matter involvement may present as a diagnostic dilemma, with HIE, hyperammonemia, seizures and encephalitis being close imaging differentials. The thalami, cerebellum and brainstem have slightly lower energy requirements and may be spared from the effects of hypoglycaemia, a feature which may help differentiate from HIE. In all situations, the clinical context and biochemical findings must be reviewed carefully to arrive at the correct diagnosis [16, 17]. Follow-up imaging plays a crucial role in prognostication. Patients with focal involvement of the splenium or internal capsules show good prognosis, whereas, insults to the neostriatum and diffuse cortical involvement often result in poor outcomes [16].

## Metabolic causes: hyperammonaemia

Ammonia is predominantly produced in the gastrointestinal tract as a by-product of amino acid metabolism, purine-pyrimidine cycle and the action of gut flora [18]. The normal blood ammonia concentration is < 35 μmol/L [19]. It is normally metabolised by the liver (urea cycle). This role is taken over by the kidneys, brain and skeletal muscles in hepatic failure [18, 20]. This alternate metabolism is inefficient, increasing the brain-blood ammonia ratio (normally in the order of 2) up to fourfold in liver failure [21]. Within the brain, astrocytes rapidly metabolise ammonia and glutamate to glutamine; however, this is physiologically costly, leading to increased cellular osmolarity and astrocyte damage. This further provokes inflammatory cascades and disrupts autoregulation, all contributing to cerebral oedema [20, 22].Other important causes of non-hepatic hyperammonaemic encephalopathy include drugs (valproate, barbiturates), adult-onset citrullinemia, surgery (e.g. ureterosigmoidostomy), parenteral nutrition and Reye syndrome [20, 22]. Patients with acute hyperammonaemic encephalopathy usually present with progressive drowsiness, seizures and coma. Prolonged hyperammonaemia can lead to severe brain injury, death or long-term sequelae such as intellectual impairment [20, 22].

**Fig 6:**
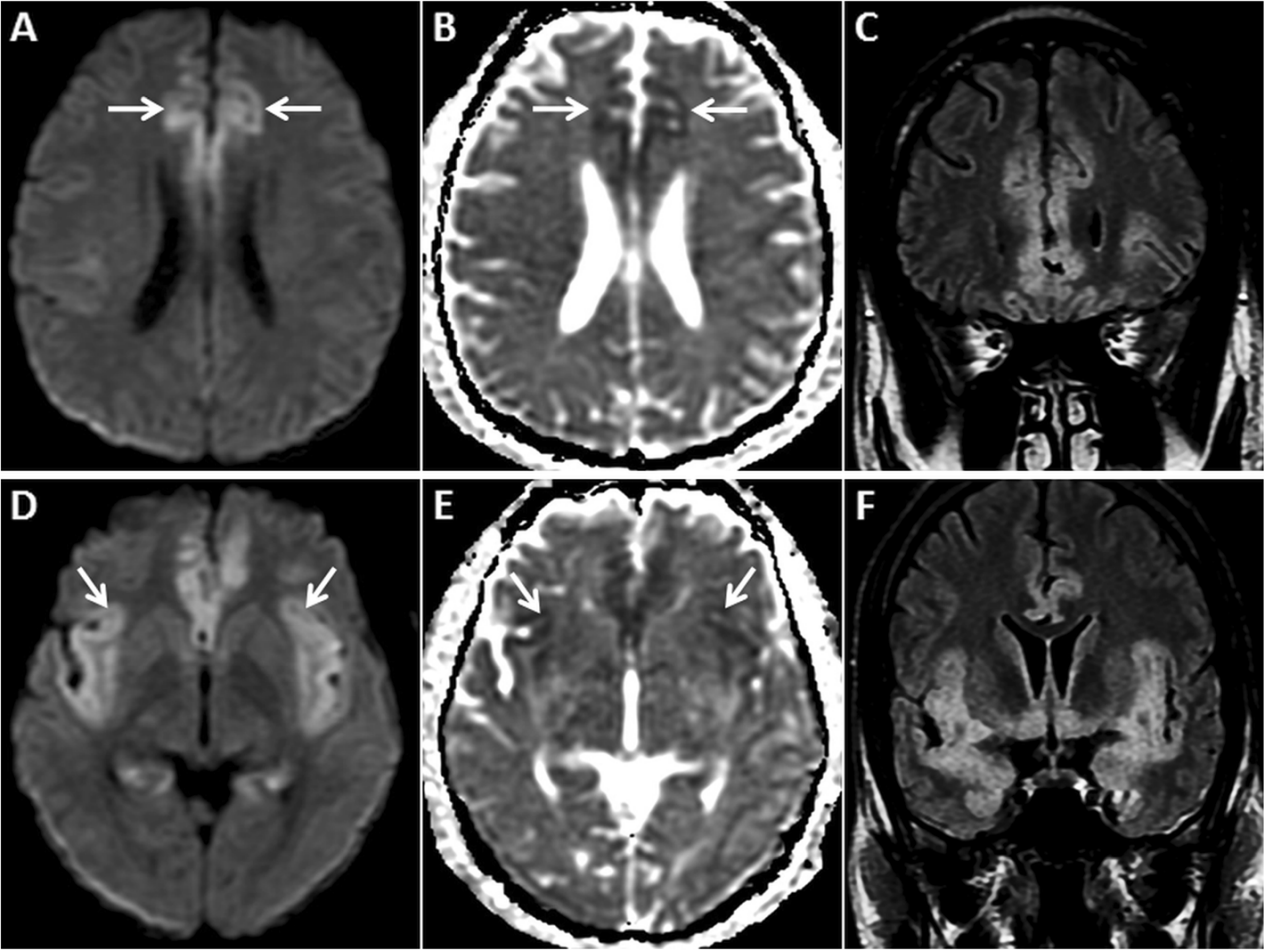
MRI of the brain in a 26-year-old female patient brought unconscious following valproate overdose. Symmetric GRD is seen in the cingulate gyri (arrows) on axial DWI (**a**) and ADC image (**b**). GRD is also seen in the insular cortices (arrows) on axial DWI (**d**) and ADC image (**e**). Coronal FLAIR images (**c**, **f**) reveal hyperintense cortical swelling in these regions, along with similar signal changes in bilateral mesial temporal lobes. Laboratory tests confirmed the diagnosis of hyperammonaemia

There are very few studies describing early imaging findings of acute hyperammonaemic encephalopathy in adults [21–23]. Rosario et al. and Takanashi et al. in independent studies have described cortical restricted diffusion in hyperammonaemia. In their studies, symmetric involvement of the insular cortex and cingulate gyrus was a common feature [22, 23] (Fig. 6). Restricted diffusion was also associated with T2/FLAIR hyperintensity. Occipital and perirolandic cortical involvement was also described [20, 21]. Bi-thalamic involvement may be seen [20, 22].

If treatment is administered in the acute phase, the signal changes may be potentially reversible. Parenchymal enhancement has been poorly studied; the limited studies that described contrast administration during imaging did not detect enhancement. Over time, pseudonormalisation of ADC signal has been described with increasing FLAIR hyperintensity. These changes occur approximately over 8 days after peak plasma levels of ammonia [20, 22].

## Infections: Cerebritis

Cerebritis is the primary manifestation of brain parenchymal infection [24, 25]. Infection can result from haematogenous dissemination or contiguous spread from infected paranasal sinuses, mastoid air cells or meninges [25, 26]. Neuroparenchymal infection passes through four pathological stages: (1) early cerebritis, (2) late cerebritis, (3) early capsular and (4) late capsular. The stage of cerebritis lasts for 10 to 14 days, characterised by ill-defined parenchymal softening with necrosis, oedema, vascular congestion and foci of haemorrhage. There is no liquefaction in this stage. If untreated, these changes eventually progress to frank abscess formation [27]. Patients with cerebritis may clinically present with headache, fever or altered mental status with or without associated sinusitis/discharging ear, etc. [25, 26].

**Fig. 7:**
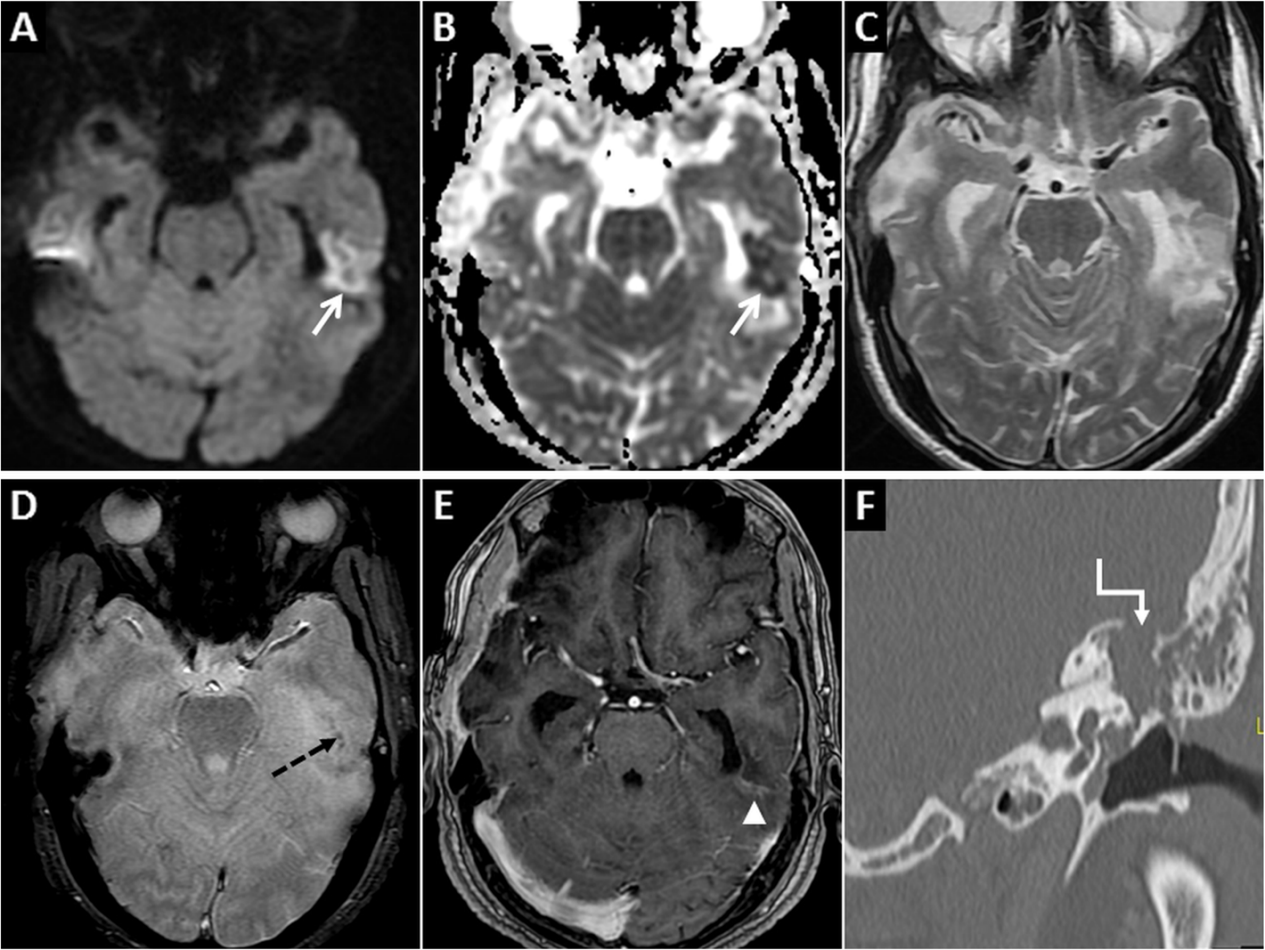
Pre and post-contrast MRI of the brain in a 70-year-old male patient who presented with altered mental status and discharging left ear. Localised GRD is seen in the posterior part of the left inferior temporal gyrus (arrows) on axial DWI (**a**) and ADC image (**b**) along with surrounding vasogenic oedema on axial T2W image (**c**). There are tiny haemorrhages (dashed arrow) in this lesion on axial GRE image (**d**) and subtle gyriform enhancement (arrowhead) on post-contrast axial T1W image (**e**). Coronal reconstruction of subsequent high-resolution CT of the left temporal bone (**f**) reveals extensive left otomastoiditis with a large bony defect in the left tegmen tympani (stepped arrow). Imaging findings are in keeping with complicated left otomastoiditis leading to cerebritis

At MRI, cerebritis appears as a poorly demarcated focal parenchymal/cortical lesion showing T1 hypointensity and T2/FLAIR hyperintensity. GRD encountered in this phase is due to high cellularity/infiltration of neutrophils, ischaemia and cytotoxic oedema (Fig. 7). Faint/subtle gyriform contrast enhancement and haemorrhages may occur at this stage [24, 25, 28]. Proximity of this cortical lesion to a known diseased site like an infected sinus or mastoid can aid in the diagnosis. If untreated, the stage of cerebritis progresses to abscess formation characterised by a central necrotic core with T2W hyperintensity, central restricted diffusion and peripheral rim enhancement [24].

## Infections: herpes encephalitis

The herpes family consists of double-stranded DNA viruses. Of the eight known Herpesviridae, Herpes simplex-1 virus encephalitis (HSVE) remains a significant cause of worldwide sporadic encephalitis in adults. Primary HSV1 infection affects the oral/nasopharyngeal mucosa and reaches the brain along the branches of the trigeminal nerve. The viral gene remains dormant in the trigeminal ganglion to reactivate in the event of immunosuppression. Viral tropism due to reactivation primarily affects the orbitofrontal and mesiotemporal lobes (limbic system). Cytotoxic injury of the cortex and grey matter structures are key features of viral encephalitides [29, 30].

**Fig. 8:**
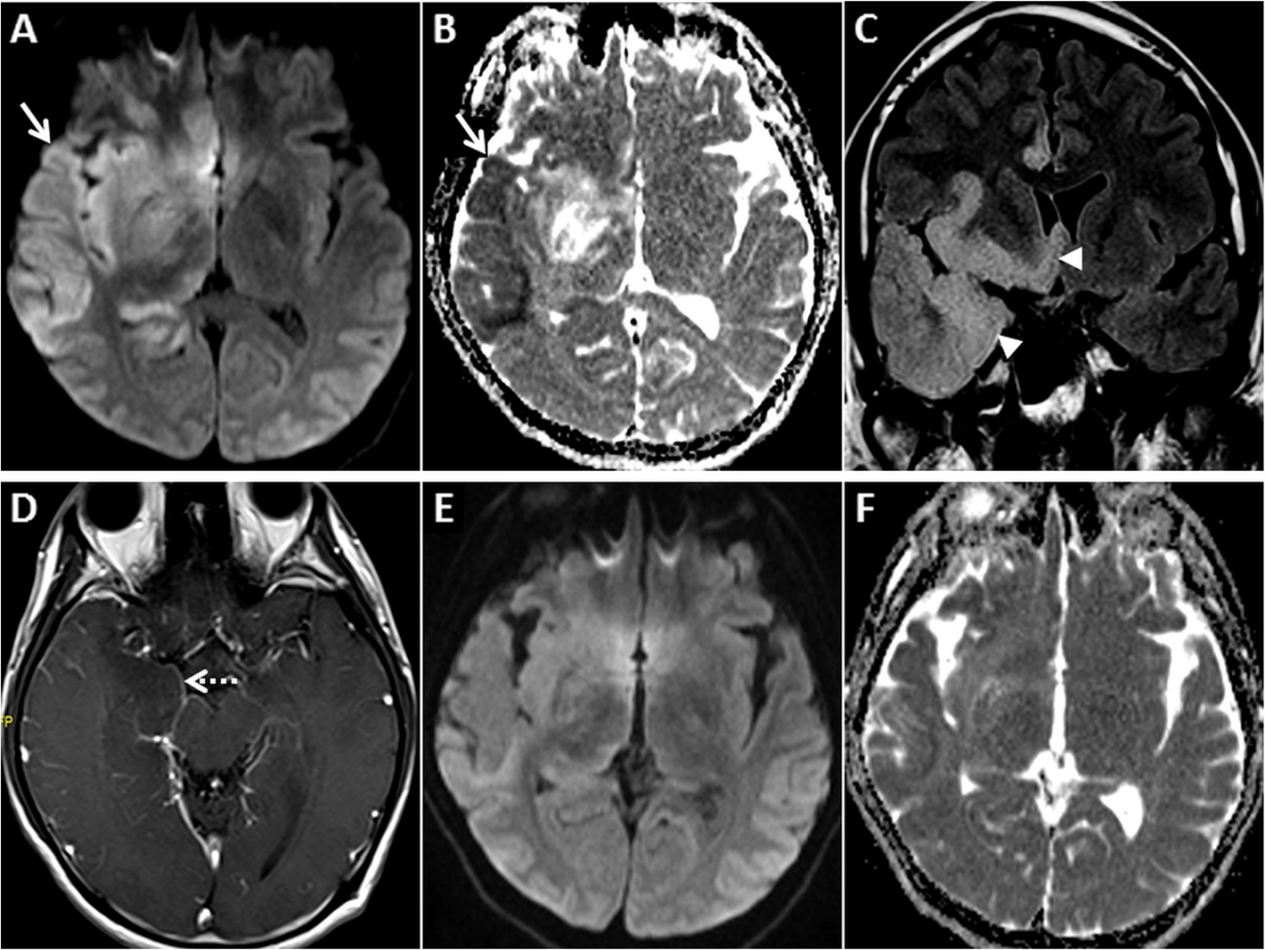
Pre- and post-contrast enhanced MRI of the brain in a 36-year-old male patient with HSVE. GRD is noted in the right temporal lobe (arrow) on axial DWI (**a**) and ADC image (**b**). Also note DWI hyperintensity in the right insula and cingulate gyrus (**a**, **b**). Coronal FLAIR image (**c**) reveals hyperintense signal and cortical swelling in the right inferior frontal and mesiotemporal regions (arrowheads). Post-contrast axial T1W image (**d**) shows smooth leptomeningeal enhancement over the right temporal cortices (dashed arrow). Post-treatment follow-up MRI shows significant resolution of previously seen GRD and cortical swelling on axial DWI (**e**) and ADC image (**f**)

Patients with HSVE typically present with fever, headache, altered mental status, seizures and focal neurological deficits. CSF typically shows pleocytosis, elevated protein and normal glucose. False-negative polymerase chain reaction test for HSV may occur early in the disease. Early clinical and imaging diagnosis and prompt treatment with acyclovir is crucial. MRI is the most sensitive imaging modality, especially in early stages of the disease. Classic imaging features include DWI hyperintensity and T2/FLAIR hyperintensity in the mesiotemporal and orbitofrontal lobes and insula (Fig. 8). Findings are typically bilateral and asymmetric but may be unilateral in the early stages. Extensive cortical swelling, vasogenic oedematous changes, haemorrhagic changes, mass effect, parenchymal and leptomeningeal enhancement are known features of HSVE [29–31]. Isolated hippocampal involvement may be more common in immune-mediated limbic encephalitis or post-ictal conditions than in HSVE [29]. Untreated cases show fulminant parenchymal necrosis, haemorrhages and are often associated with high mortality rates ranging between 50 and 70%. Atrophy and encephalomalacia of affected regions are typical sequelae of HSVE [29–31].

## Infections: Creutzfeldt-Jakob disease

Creutzfeldt-Jakob disease (CJD) is a transmissible encephalopathy, grouped under prion (infectious proteins without nucleic acid) diseases, characterised by spongiform brain degeneration. Most cases are sporadic (sCJD), while 10% are hereditary. When infected, the healthy cellular prion proteins (PrP^C^) are converted to scrapie isoforms (PrP^Sc^), which cause neuronal death [32–34].

Neurological symptoms include rapidly progressing dementia, myoclonus, pyramidal or extrapyramidal features, and akinetic mutism. Death occurs within a year of onset [32]. Periodic sharp-wave complexes and 14-3-3 proteins may be encountered on EEG and in the cerebrospinal fluid (CSF), respectively [35]. MR signal changes especially on DWI (sensitivity approximately 100%) precede clinical symptoms and EEG/CSF changes, making it the primary modality in early diagnosis [35, 36].

**Fig. 9:**
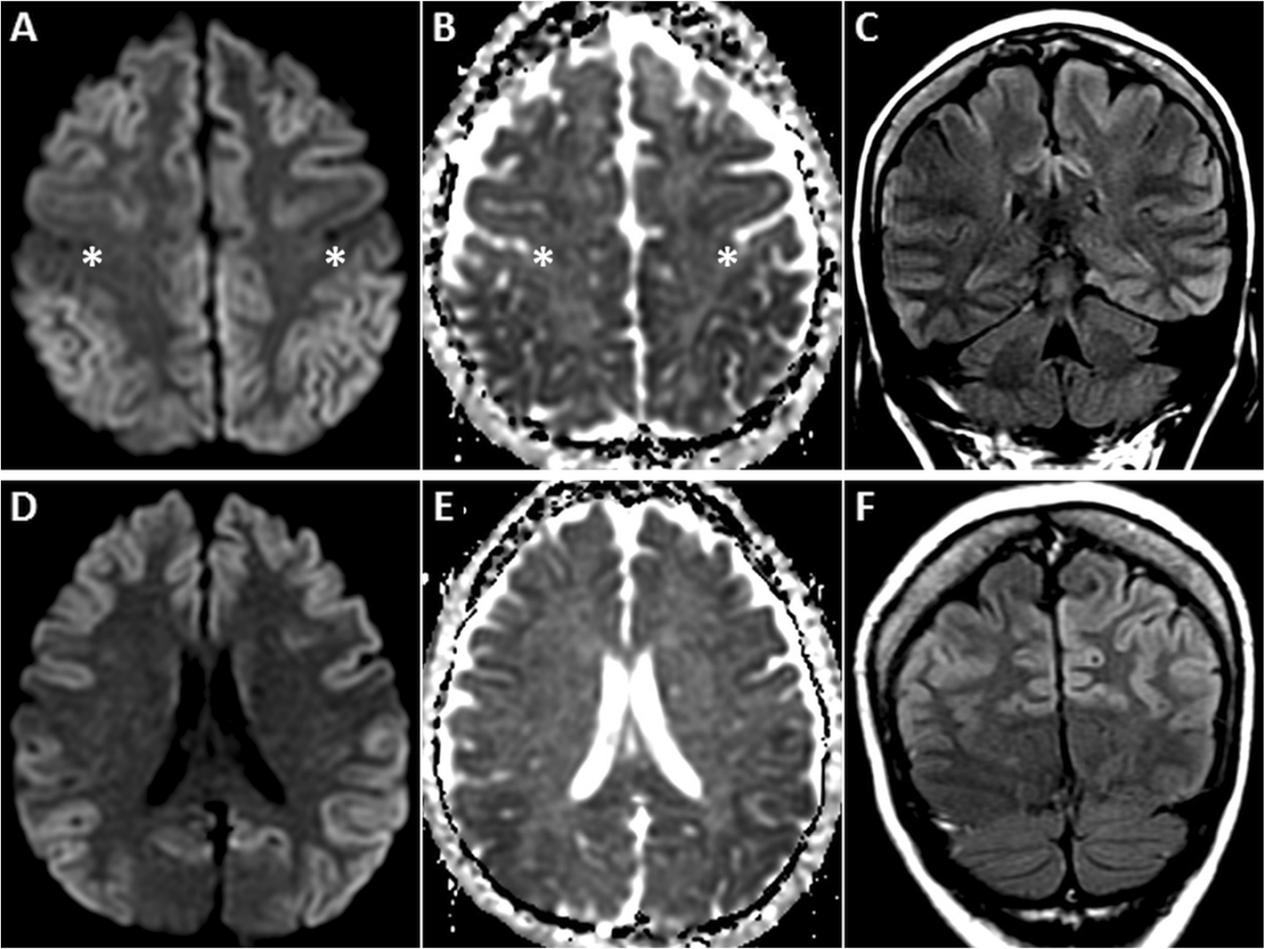
MRI of the brain in a 58-year-old female patient who presented with rapidly progressive dementia due to sCJD. Symmetric GRD is seen in the cerebral cortices on axial DWI (**a**, **d**) and ADC images (**b**, **d**, **e**) with sparing of the perirolandic regions (asterisks in **a**, **b**). Coronal FLAIR images (**c**, **f**) shows hyperintense signal in the affected cortices

**Fig. 10:**
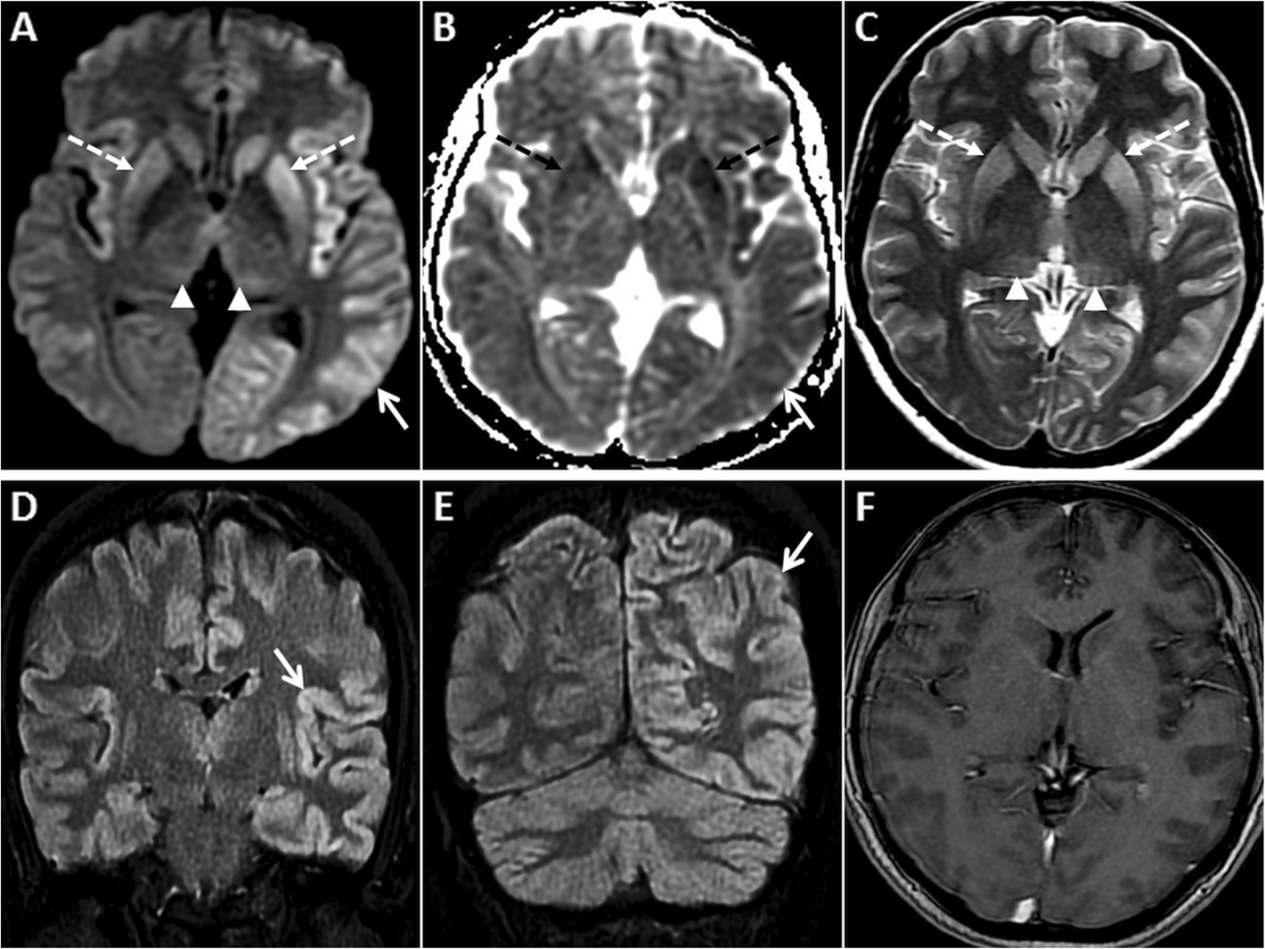
Pre- and post-contrast MRI of the brain in a 55-year-old male patient who presented with rapidly progressive dementia due to sCJD. GRD is seen in the left temporo-parieto-occipital region (arrows) and in the insular regions on axial DWI (**a**) and ADC image (**b**). Nearly symmetric restricted diffusion is also seen in the basal ganglia (dashed arrows) along with mild DWI hyperintensity in the pulvinar nuclei (arrowheads). Axial T2W image (**c**) shows T2 hyperintensity in the basal ganglia (dashed arrows) and pulvinar nuclei (arrowheads). Sequential anteroposterior FLAIR images (**d**, **e**) demonstrate the extent of the cortical signal abnormality (arrows). No enhancement is seen in these regions on axial post-contrast T1W image (**f**)

The typical MR imaging pattern seen in sCJD consists of DWI hyperintensity in the cerebral cortex and the basal ganglia. The insular cortex and the cingulate gyri (limbic system) as well as the superior frontal gyri are commonly involved. The perirolandic region is usually spared [36] (Fig. 9). Symmetric hyperintense DWI and T2W/FLAIR signals in the pulvinar nuclei of the thalami (“pulvinar” or “double hockey stick” sign) which are the hallmark of variant CJD (vCJD), are rare in sCJD (Fig. 10). Post-contrast enhancement does not occur [37]. With disease progression, the cortical signal abnormality may decrease or even disappear on DWI, owing to neuronal death and atrophy [36].

## Genetic: MELAS

MELAS (mitochondrial myopathy, encephalopathy, lactic acidosis, stroke-like episodes) is a multi-system disorder grouped under mitochondrial metabolic diseases. It is caused by the inheritance of a mutated mitochondrial DNA in the maternal line. This abnormal DNA causes poor energy production, microvascular angiopathy and nitric oxide deficiency. Thus, high-energy requiring tissues, such as the brain and skeletal muscles, are affected [38].

Symptoms usually manifest by the age of 40. These include delayed and stunted growth, headache, recurrent stroke-like episodes, sensorineural hearing loss and cortical blindness [39, 40]. Genetic analysis or muscle biopsy to identify ragged red fibres using modified Gomori trichrome or succinate dehydrogenase stains are essential for a definitive diagnosis [38].

**Fig. 11:**
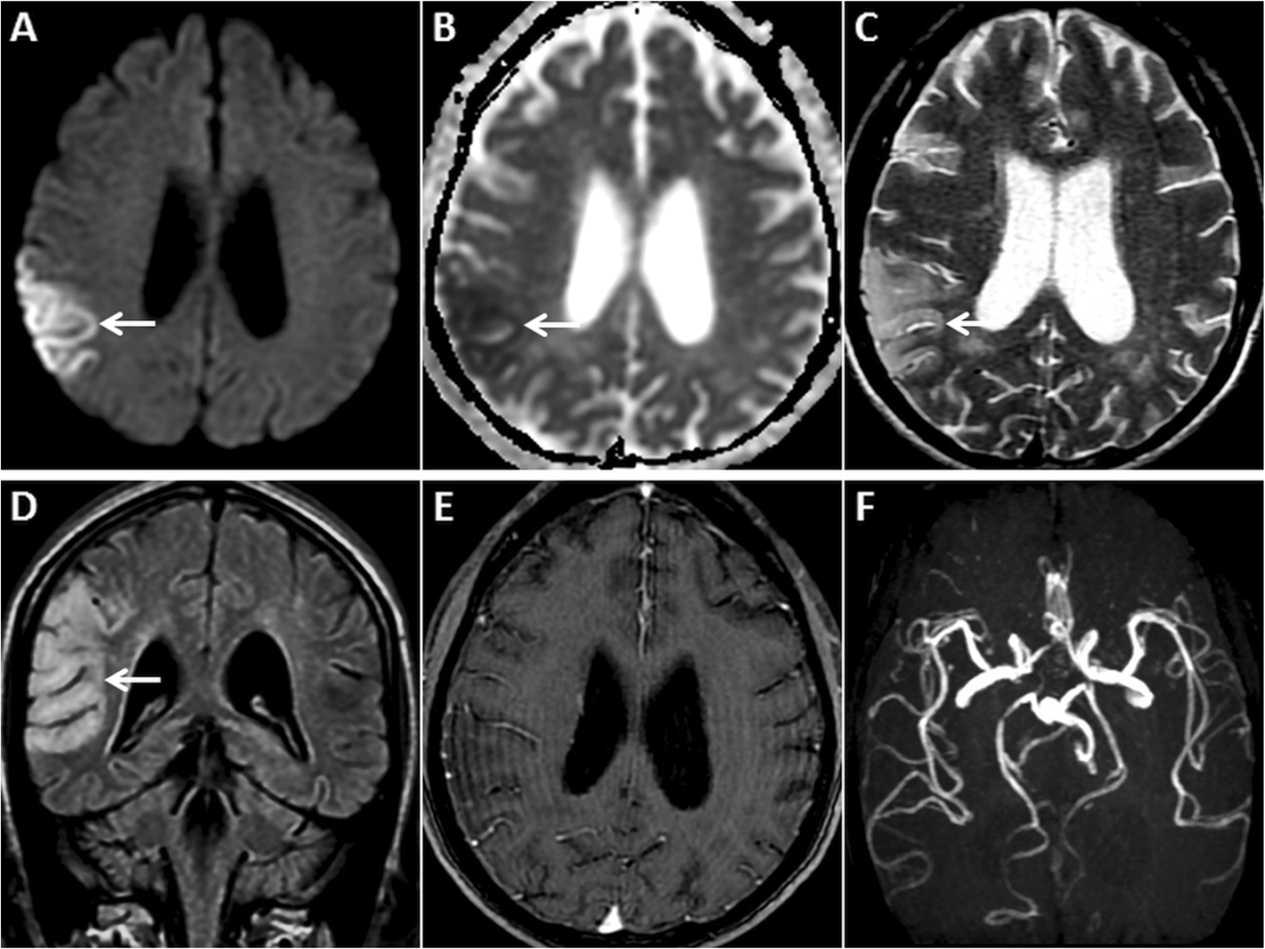
Pre- and post-contrast MRI of the brain in a 45-year-old male patient with proven MELAS on muscle biopsy. GRD is observed in the right parietal cortex (arrows) on axial DWI (**a**) and ADC image (**b**). Associated gyral swelling and hyperintense signal (arrows) is seen on axial T2W (**c**) and coronal FLAIR image (**d**). No enhancement is seen in this region on post-contrast axial T1W image (**e**). Reconstructed maximum intensity projection MRA image of Circle of Willis (**f**) confirms no large vessel occlusion

On MRI, MELAS shows a predilection for the parietal, temporal and occipital cortices, not conforming to vascular territories. Within 24 h of a stroke-like episode, affected areas demonstrate GRD with T2W prolongation (because of cytotoxic oedema from energy insufficiency) [38, 41] (Fig. 11). Angiograms are usually unremarkable. Lesions show a migratory, waxing and waning pattern with DWI changes paralleling the disease course. They resolve with clinical improvement only to recur in new locations with new symptom onset. Contrast enhancement may occur, and MR spectroscopy may show an elevated lactate peak at 1.33 ppm [38, 42]. Extensive petechial haemorrhages along the involved cortices have also been reported [42]. A compliation of the information from the references and the discussion from the various sections in the manuscript is summarised in Table 1.

**Table 1:** Pattern-based approach to GRD

Aetiology	Pattern	Cortical predilection	T2 signal	GRE susceptibility	PGE	Other involvement
HIE	B/L, symmetric	Perirolandic, occipital cortex	**+**	**+/−**	**−**	Basal ganglia, thalami, cerebellum
Post-ictal	U/L or B/L, diffused or localised	Any lobe, **+/−** hippocampus	**++**	**−**	**+/−**	**+/−** Splenium
Hypoglycaemia	B/L, symmetric > asymmetric or U/L	Parieto-occipital, **+/-** hippocampus	**++**	**−**	**+/−**	**+/−** basal ganglia, splenium
Hyperammonaemia	B/L, symmetric	Insula, cingulate gyrus	**++**	**−**	**−**	**−**
Cerebritis	U/L, localised	Temporal, frontal	**++**	**+/−**	**+**	Sinusitis/mastoiditis
Herpes	Initially U/L, progresses to asymmetric B/L	Orbitofrontal, mesiotemporal, insula	**++**	**+**	**++**	Leptomeningeal enhancement
CJD	Diffuse, symmetric/asymmetric	Insula, cingulate gyrus, superior frontal gyrus	**+**	**−**	**−**	Pulvinar
MELAS	U/L, migratory, waxing-waning	Parietal, temporal, occipital	**++**	**+/−**	**+/−**	**−**

*GRE* gradient echo sequence, *PGE* post-gadolinium enhancement

## Conclusion

Restricted diffusion involving the cerebral cortex is commonplace, especially in a high volume neurology/neuroimaging setting. Of the differentials, vascular thrombo-occlusion is the most common aetiology. However, occasionally, GRD may be caused by non-stroke conditions like metabolic encephalopathies, hypoxia, seizures and infections. These non-stroke conditions may be challenging to diagnose. Awareness of the various differentials and thorough knowledge of imaging features in conjunction with accurate clinical findings helps to clinch the accurate diagnosis and guide appropriate management.

## Data Availability

Not applicable

## Abbreviations

ADC: Apparent diffusion coefficient
ATP: Adenosine triphosphate
B/L: Bilateral
CJD: Creutzfeldt-Jakob disease
CSF: Cerebrospinal fluid
DWI: Diffusion-weighted imaging
EEG: Electroencephalogram
GRD: Gyriform restricted diffusion
GRE: Gradient echo sequence
HIE: Hypoxic-ischaemic encephalopathy
HSVE: Herpes simplex-1 virus encephalitis
MELAS: Mitochondrial myopathy, encephalopathy, lactic acidosis, stroke-like episodes
PGE: Post-gadolinium enhancement
sCJD: Sporadic Creutzfeldt-Jakob disease
U/L: Unilateral
vCJD: Variant Creutzfeldt-Jakob disease
